# Correction: Adhesives free bark panels: An alternative application for a waste material

**DOI:** 10.1371/journal.pone.0315050

**Published:** 2024-12-03

**Authors:** The PLOS One Staff

There is an error in the caption for Fig 5. The line thickness equals interquartile range should have been from 25^th^-75^th^ percentile. Please see the correct, complete caption of Fig 5 here.

**Fig 5:**
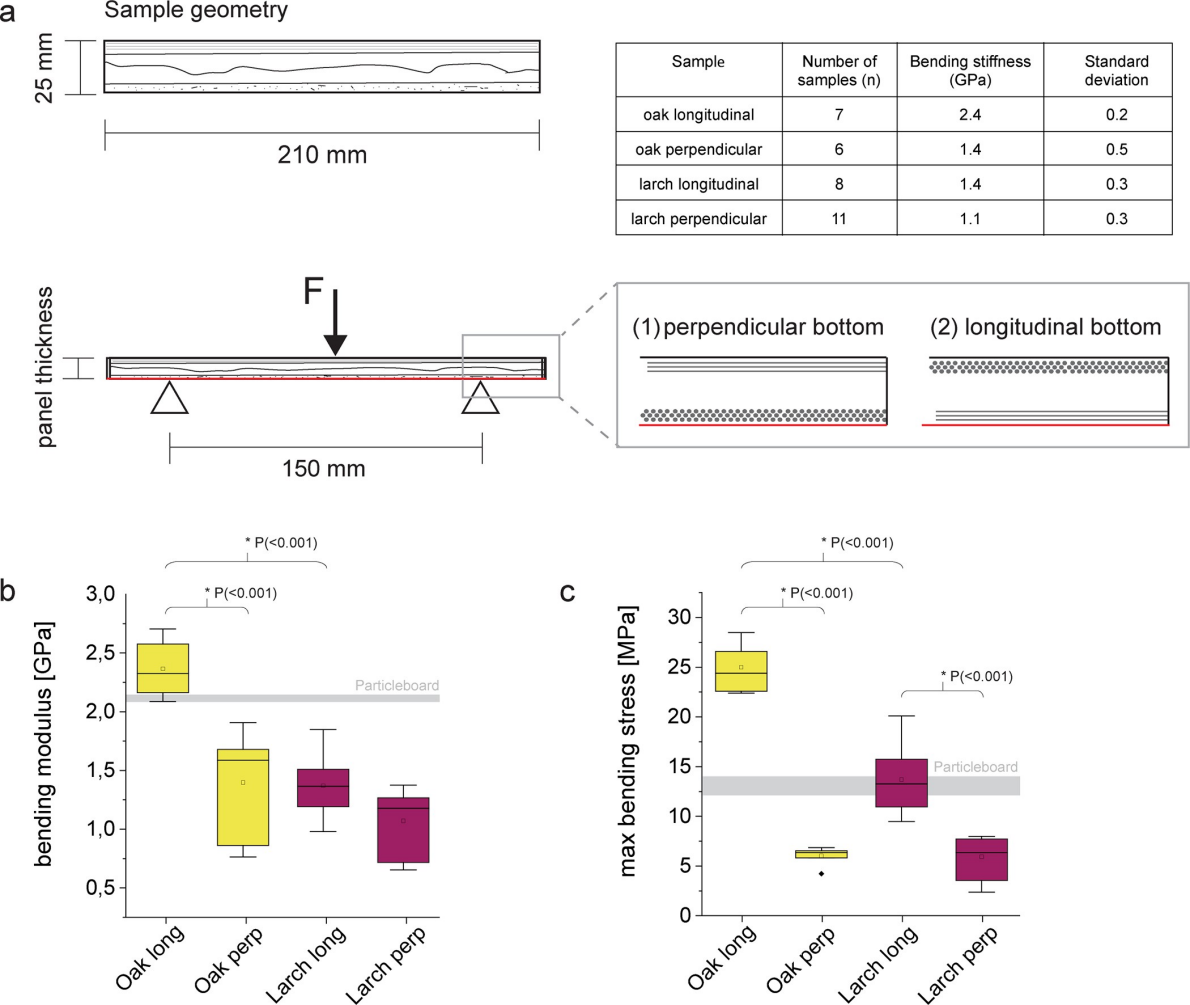


Mechanical data of three-point-bending experiments (a) samples with a length of 210 mm and a width of 25 mm were cut and tested with a test span of 150 mm. Sample thickness corresponded with panel thickness, the orientation of the fibers at the bottom was either perpendicular (1) or longitudinal (2). (b) bending modulus of the tested samples (c) max bending stress of the specimens. Boxes show 25th–75th percentile, small rectangle in box is the mean and the line the median of the samples, stars correspond to outliers. Gray line in the diagrams shows bending modulus of the tested particle board with a density of ~ 0.7 g/cm3 (line thickness equals interquartile range from 25th– 75th percentile).

The publisher apologizes for the error.
