# Supplementary material for: Adhesives free bark panels: An alternative application for a waste material
Source: PLoS One. 2023 Jan 23;18(1):e0280721. doi: 10.1371/journal.pone.0280721 (PMC9870117; doi:10.1371/journal.pone.0280721)
Supplement: Attachment — Submitted filename: response to reviewers.docx [file pone.0280721.s001.docx]

**Response to Reviewers' comments:**

*Dear Reviewers,*

*First of all we wish to thank you for your time and efforts to read and carefully review our manuscript. In the following we will respond to raised questions, concerns and comments point by point. The changes in the manuscript are in red, a few corrected typos are not specifically marked.*

*Sincerely yours,*

*Charlett Wenig and Michaela Eder on behalf of all authors*

Reviewer #1: Wenig et al provide a nice comperehensive study in which the properties of adhesive free bark panels from bark of different origin are investigated. The study is relevant in terms of the use of a waste material in bulk material application avoiding potentially toxic adhesives that are commonly used. The findings are clearly presented and the conclusions are sound. Publication in PLOS ONE is recommended and only some minor recommendations for improvements are provided:

Introduction:
-The author refer to studies performed in the mid-20th century but then give citations for manuscript in the 21st century. *Thank you for pointing this out! We removed the text relating to the 20^th^ century since the more recent studies are more relevant.*
- When refering to the study by Burrows et al it seems relevant to mention the type of bar used (Douglas Fir). *We agree that the bark type is important and added the information*

Results and discussion:
- It took me a bit to figure out what the two pictures on the bottom of Figure 2a meant. I recommend adding a label *we added a label and hope that it is clearer now*
- Also I recommend to add enlargement of the side view for the panels to clearly show that the boundry lines are visible (now this only is the case for Oak) *the side view is now enlarged*
- Figure 3 seems to be placed in the wrong section *we moved it to the appropriate section*
- Table 1 is not refered to in the section it is placed in *we changed this*
- Table 1 column 4 should be density *thank you, we corrected the column heading*

References:
- Please check formatting, some could not be easily traced. *We checked formatting*
- What is the footnote at the end of the references for? *Here a formatting error occurred. The footnote is now at the end of page 2 in order to provide additional information related to historic bark use and its documentation (lines 49-51).*

Reviewer #2: The work is well written and relevant. Correct methodology. The results were analyzed correctly. Some recommendations: objetives at the end of the introduction and put specific objetives. *We added some specific objectives towards the end of the introduction*

Methodology: cite the age, diameter and height of the trees. Reason for choosing species. *The species were selected according to their availability in the vicinity of our research institution since one of the goals was to work with local trees. Since the aim of the present work was to explore the potential of local tree barks as a raw material for panels, age, diameter and height were not recorded as well as location of the bark within the tree. However, we observed a large variation in bark thickness, curvature etc both within and between trees. This is certainly a topic of future research and has now been included in the text.*

Results and discussions: to discuss the relationship of the influence of tree diameter on debarking. Mention the thickness of the barks. Amount of phloem and periderm. Show the cells of the barks. *Thank you for your comment and your mentioned aspects are certainly interesting questions and should be addressed in the future. As written in the previous answer, the main goal of this work was a general exploration about the feasibility of using bark as a raw material to create panels. The µCT scans provide information about the cells in the barks, a detailed analysis phloem and periderm amount would require an elaborate sampling considering tree heights, ages, etc. and was beyond the scope of the present work.*
